# Associations between Antibacterial Treatment and the Prevalence of Tail-Biting-Related Sequelae in Danish Finishers at Slaughter

**DOI:** 10.3389/fvets.2017.00182

**Published:** 2017-11-01

**Authors:** Mette Fertner, Matt Denwood, Anna Camilla Birkegård, Helle Stege, Anette Boklund

**Affiliations:** ^1^Department of Veterinary and Animal Sciences, University of Copenhagen, Frederiksberg, Denmark; ^2^National Veterinary Institute, Technical University of Denmark, Kongens Lyngby, Denmark

**Keywords:** slaughter remarks, pigs, swine, meat inspection, hierarchical model, antibiotic use, antimicrobial use, abattoir

## Abstract

Secondary infections as a result of tail biting cause substantial economic losses in pig production and are a subject of concern for animal welfare. The use of first-choice antibacterial agents in the treatment of tail biting in finishing pigs is hypothesized to be negatively correlated with the development of systemic infection. This would be expected to reduce the prevalence of *post-mortem* pyemic sequelae (such as osteomyelitis and abscesses) in finishers with tail-bite lesions. We performed a register-based study that included three Danish databases, holding information on the purchase of antibacterials at herd level (VetStat), herd demographics (Central Husbandry Register), and relevant observations at slaughter (meat inspection data). We included all finishers from indoor production finisher herds that met the inclusion criterion of at least one slaughtered finisher with a recorded tail-bite observation during 2015 at the single largest Danish abattoir. The final dataset held 1,070 herds with one or more tail-bite observations, from which 14,411 of 2,906,626 finishers (0.50%) had an individual record of a tail bite. Within this group of finishers with tail-bite observations, the recorded tail-biting-related sequelae included osteomyelitis (8.1%), abscesses in the hindquarters (10.5%), abscesses in the forequarters (2.3%), abscesses in the mid-section of the carcass (2.9%), abscesses in the limbs (2.4%), and chronic arthritis (0.5%). Due to a high-herd prevalence (>25%), osteomyelitis and abscesses in the hindquarters were selected for further analysis. The occurrence of osteomyelitis and hindquarter abscesses in individual finishers with tail-bite observations was described using a generalized linear mixed effects model with binomial response and logit link. Herd was included as a random effect, while herd size and various antibacterial treatments were tested for inclusion in the model as fixed effects. The final models indicated a significant association between herd size and both osteomyelitis (*p* = 0.014) and hindquarter abscesses (*p* < 0.001), with larger herds (2,001–12,000 registered finisher pigs) showing a reduced risk. Further, a negative association was found between the occurrence of hindquarter abscesses and the use of oral pleuromutilin (*p* = 0.022). The significant association with herd size highlights the potential importance of management factors in reducing the occurrence of tail-bite lesions in finishing pigs.

## Introduction

Tail biting is of substantial economic importance in industrial pig production due to the potential for secondary infection, reduced performance, euthanasia, and the condemnation of carcasses from slaughter pigs (1–4). Osteomyelitis, embolic pneumonia, abscesses, and arthritis have all been found to be associated with tail lesions in slaughtered pigs (1, 3).

A lesion in the tail enables pyogenic bacteria from the environment and skin to enter the lymphatic drainage system leading to the sacral lymph nodes. From here, pyogenic bacteria may spread locally by retrograde dissemination through lymph or blood to cause osteomyelitis in the pelvis or tail. Additionally, pyogenic bacteria may enter the blood stream (pyemia) and cause sequelae in other parts of the body (5, 6). A Danish study on carcasses with a tail lesion performed bacteriological culture from lymph nodes and abscesses and found *Trueperella pyogenes* and/or *Fusobacterium necrophorum* to be the primary pathogens involved [found in 84% of the cases (7)]. According to the Danish circular on meat inspection (6), recording of a tail-biting observation in the abattoir requires a subsequent pyemia examination of the carcass. This means that pigs with tail-bite lesions are more likely to receive a registration of pyemiac sequelae compared with pigs without a tail bite, due to the selection bias associated with risk-based surveillance.

Clinical signs of tail biting have previously been found in 1.26% of Danish finishers (8), while 0.84% of 9,481 Danish slaughter carcasses were found to have tail lesions (7). Severe cases of tail biting may result in death or euthanasia, or injured tails may heal before slaughter (9). This would lead to a reduced number pigs with tail-bite lesions registered at slaughter compared with the prevalence observed clinically (10).

Tail biting mainly arises in the early finishing period (30–60 kg), although it may also be seen in other age groups (2, 11, 12). Tail biting may occur around the age of puberty, where the interest in the tail region of other animals increases (3). This behavior may be triggered by a number of external risk factors such as the absence of rooting material, a high-stocking rate, large herds, or fluctuations in ventilation, temperature, and feeding (2). To reduce the risk of tail biting, tail docking is performed widely in the industrialized pig industry (10), although legislation from the European Union discourages routine tail docking (13). In Denmark, tail docking without long-lasting analgesia is only permitted in the second to fourth day after birth, and no more than half of the length of the tail should be docked (14). In addition, this practice is only permitted in herds that can document that tails will have lesions if docking is not performed (14). As this is the case for the vast majority of Danish production herds, docking is widely used in industrialized pig production in Denmark. When tail biting occurs in the pen, the farmer is advised to identify and remove the biter, and to further isolate and treat finishers with tail lesions (11). According to the official treatment recommendations from the Danish Veterinary and Food Administration, the first-choice treatment for pigs with tail-bite lesions is either parenteral benzylpenicillinprocain or oral pleuromutilin (15). However, tetracycline may also be prescribed for treatment by some veterinarians based on tradition. In Denmark, antibacterials used for veterinary purposes require a prescription and are registered in the national database, VetStat (16).

A previous study indicated that antibacterial treatment of pigs with tail-bite lesions did not prohibit the spread of infection in all cases due to a higher prevalence of abscesses in pigs with tail-bite lesions compared with those without (17). However, proper treatment of tail biting may reduce the risk of developing systemic infection and thus reduce the prevalence of pyemic sequelae found *post-mortem*. The objective of this study was therefore to assess whether the quantity of antibacterial purchased for the individual herd was associated with the prevalence of pyemic sequelae registered at the time of slaughter in finishers with tail-bite lesions from indoor commercial finisher herds.

## Materials and Methods

### Study Design

The study was based on data from three Danish registers, relating to: antibacterial purchases (VetStat), herd demographics [Central Husbandry Register (CHR)], and meat inspection. We included all commercial indoor finisher herds that had delivered at least one finisher (<130 kg) with a remark relating to an injured tail for slaughter to the largest Danish abattoir in 2015. Registrations on antibacterial purchases for these herds for the period January 1 to December 31, 2015 were retrieved from VetStat. CHR data extractions from December 31, 2014 to December 31, 2015 were compared. Herds with any changes in herd size between these two dates were excluded.

### Slaughter Remarks

Remarks relating to tail lesions and four potential tail-bite-related sequelae (osteomyelitis, arthritis, abscesses, and embolic pneumonia) were identified. Meat inspection codes were recorded in accordance with Danish legislation (6), where abscesses were split into four sub-categories related to the part of the carcass that was involved: forequarters, mid-section, hindquarters, and limbs. The two lesions indicative of a tail bite (i.e., “infected tail lesion” and “localized tail lesion”) were aggregated into a single category, which was used in this study to define the individual pigs for which tail biting was recorded.

### Antibacterial Use

Information on antibacterials purchased for finishers in the studied herds during 2015 was retrieved from VetStat on January 9, 2017. Antibacterials were categorized according to active substance, based on the Anatomical Therapeutic Classification system and further subdivided into oral or parenteral administration as registered for each product in VetStat. Antibacterials recommended for use in pigs with tail-bite lesions were retained as separate categories, while all other substances were grouped into a single category. As a result, the final antibacterial categories covered narrow-spectrum penicillin (parenteral), pleuromutilin (oral), tetracycline (parenteral), tetracycline (oral), other antibacterials (parenteral), and other antibacterials (oral).

All antibacterial amounts were quantified as animal daily doses (ADDs) and subsequently combined with CHR data to obtain a quantity of ADDs per 100 finishers per day (ADD_50_/100 finishers/day). This unit approximates the percentage of pigs that are treated daily within the herd (18).

### Study Population

**Figure 1:**
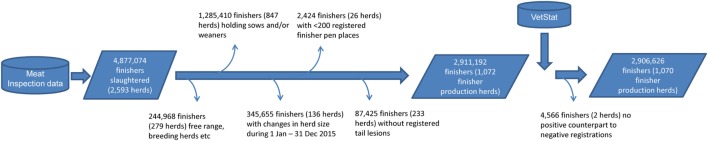
Flow diagram of the selection process of 1,072 Danish finisher production herds (2,906,626 slaughtered finishers), with a minimum of one finisher with a tail-bite lesion at the time of slaughter in the largest Danish abattoir in 2015.

The study population was selected as presented in Figure 1. During 2015, a total of 4,877,074 finishers originating from 2,593 herds were slaughtered in the largest abattoir in Denmark. For the study, we excluded finishers originating from herds other than production herds (244,968 finishers from 279 free range herds, breeding herds, etc.), herds that also contained sows and/or weaners (1,285,410 finishers, 847 herds), and herds that had changed their number of registered finishers in the CHR between December 31, 2014 and December 31, 2015 (345,655 finishers, 136 herds). In addition, herds with fewer than 200 registered pen places were excluded (2,424 finishers, 26 herds), as well as herds with no finishers registered with tail lesions (87,425 finishers, 233 herds).

For the selected herds, purchases of prescription-only drugs, as registered in VetStat, were extracted (665 registrations by feed mills, 1,542 registrations by veterinarians, and 23,795 registrations by pharmacies). Mismatches between registered animal species and age group were checked manually and corrected (three registrations) (19).

Purchases of antibacterials specifically prescribed for the relevant age group and species (finisher pigs) were selected (0 registrations by feed mills, 19 registrations by veterinarians, and 19,860 registrations by pharmacies). Negative registrations may be found in the VetStat database due to retrospective corrections of incorrectly registered purchases of drugs (19). To correct for this, we matched negative registrations with their positive counterparts and deleted the pair (214 registrations), resulting in a total of 19,665 registrations. This was not possible for two herds, so we excluded both herds from the study (4,566 finishers, 2 herds). The final dataset for further analysis therefore contained 2,906,626 finishers originating from 1,070 production herds (Figure 1).

### Statistical Analyses

Statistical models were used to quantify risk factors for the occurrence of tail biting within all finisher pigs, and for the occurrence of sequelae within individual animals with observations of tail biting. The latter analyses were restricted to sequelae that occurred in a minimum of 25% of the studied herds, and a separate generalized linear mixed model with binomial outcome and logit link was used for each. This model is equivalent to a logistic regression at individual animal level, so herd was used as a random effect in all models to control for this potential clustering. All models were implemented using the lme4 package and R version 3.3.2 (20, 21).

Potential risk factors for the occurrence of tail biting and selected sequelae were tested independently, but using the same procedure. Univariate models were used to screen each of the candidate risk factors in turn, with an inclusion criteria of *p* < 0.20 for inclusion in the full model. The final model was subsequently obtained from the full model using backward elimination based on likelihood ratio tests. All risk factors included in the final multivariate model were also evaluated for potential confounding. The candidate risk factors, which were all at herd level, were as follows: herd size, use of simple penicillin (parenteral), use of pleuromutilin (oral), use of tetracycline (parenteral), use of tetracycline (oral), use of other antibacterials (parenteral), and use of other antibacterials (oral). As less than 50% of the herds used pleuromutilin (oral), tetracycline (oral), and other antibacterials (oral), we dichotomized these three variables as “use” versus “no use” for the herd. Risk factors for simple penicillin (parenteral), tetracycline (parenteral), other antibacterials (parenteral), and herd size were each classified into three categories (low/medium/high). These categories were based on breakpoints that generated groups of approximately equal size. For risk factors including more than two categories, the null hypothesis of all categories being equal was assessed using likelihood ratio tests.

## Results

### Descriptive Statistics

The category “other antibacterials administered orally” consisted of 89% macrolide and 8% extended-spectrum penicillin, with the remaining 3% including amphenicol, colistin, lincosamide, lincospectin, and sulfamethoxazole–trimethoprim. Other antibacterials administered parenterally consisted of 61% lincosamide, 16% macrolide, and 11% pleuromutilin, with the remaining 12% including amphenicols, combination-drugs, lincomycin–spectinomycin, sulfamethoxazole–trimethoprim, and extended-spectrum penicillin.

Of the slaughtered finishers, 14,411 (0.50%) had a remark relating to a tail bite at the time of slaughter. Of these, 2,715 (19%) originated from small herds, with a median of 4 finishers with tail bites per year (range: 1–72); 6,346 (44%) originated from medium-sized herds with a median of 7 finishers with tail bites per year (range: 1–147); and 5,350 (37%) originated from large herds with a median of 12 finishers with tail bites per year (range: 1–310). The median within-herd prevalence of finishers with a remark relating to a tail-bite observation was 0.4% (range: 0.01–25%). Tail-bite-related sequelae were recorded as shown in Table 1. Osteomyelitis and hindquarter abscesses had a herd-level prevalence of over 25%, and were therefore used as outcomes for the statistical model, along with the occurrence of tail biting itself. The frequency of osteomyelitis was significantly higher in finishers with hindquarter abscesses (12.9%, 196/1,516) than finishers without these abscesses (7.6%, 980/11,915; χ^2^ test: *p* < 0.001). There were 463 (43%) herds in which osteomyelitis was recorded for at least one finisher with a tail-bite lesion (Table 1), and within these herds the herd-level prevalence of osteomyelitis was 12.5% (range: 2.4–100%) among finishers with a recorded tail-bite lesion. Similarly, hindquarter abscesses were found in 551 (51%) of the herds, in which 14.3% (range: 1.5–100%) of the finishers with a recorded tail-bite lesion had a concurrent remark of one or more hindquarter abscesses.

**Table 1:** Tail-bite-related slaughter remarks registered in 14,411 finishers with tail-bite lesions that were slaughtered in the largest Danish abattoir during 2015.

Slaughter remark	Number of finishers with tail-biting injuries	Number of herds
Osteomyelitis	1,176 (8.1%)	463 (43%)
Embolic pneumonia	197 (1.4%)	136 (13%)
Chronic arthritis	76 (0.5%)	69 (6%)
Forequarter abscesses	331 (2.3%)	234 (22%)
Mid-section abscesses	422 (2.9%)	257 (24%)
Hindquarter abscesses	1,515 (10.5%)	550 (51%)
Limb abscesses	347 (2.4%)	230 (13%)

### Modeling

Separate models were used for each of the three selected outcomes as described above. For the occurrence of tail biting among all slaughtered finishers, univariate screening (Table 2) indicated herd size, simple penicillin (parenteral), pleuromutilin (oral), other antibacterials (oral), and other antibacterials (parenteral) to be used for the full model, with all but herd size subsequently eliminated by backwards elimination. For the occurrence of osteomyelitis, univariate screening indicated herd size, other antibacterials (oral), and other antibacterials (parenteral) to be used for the full model, with other antibacterials (parenteral) subsequently eliminated by backwards elimination. For the occurrence of hindquarter abscess, univariate screening indicated herd size and pleuromutilin (oral) to be used for the full model, with both factors retained during backwards elimination. The final models therefore comprised of the following risk factors:

(1) Occurrence of tail biting among all finishers:
  - Herd size.
(2) Occurrence of osteomyelitis among finishers with tail-bite lesions:
  - Herd size.
  - Other antibacterials (oral).
(3) Occurrence of hindquarter abscesses among finishers with tail-bite lesions:
  - Herd size.
  - Pleuromutilin (oral).

**Table 2:** Univariate generalized linear mixed models with herd as a random effect and three Binomial outcomes of: (1) the occurrence of tail-bite observations among all finishers slaughtered; (2) the occurrence of osteomyelitis among those finishers with recorded tail-bite observations; and (3) the occurrence of hindquarter abscesses among those finishers with recorded tail-bite observations.

		Number (%)	Tail bite (*p*-value)	Osteomyelitis (*p*-value)	Hindquarter abscesses (*p*-value)
Total number of finishers slaughtered	1–500	196 (18)	–	–	–
	501–3,000	538 (50)			
	3,001–44,733	336 (31)			
Number of finishers with an injured tail	1–4	412 (39)	–	–	–
	4–10	283 (26)			
	11–310	375 (35)			
Herd size (number of finishers registered)	200–1,000	364 (34)	<0.001	0.02	<0.001
	1,001–2,000	454 (42)			
	2,001–12,000	252 (24)			
Antibacterial use (ADD/100 finishers/day)					
Simple penicillin (parenteral)	Low (≤0.1)	296 (28)			
	Medium (0.1–0.3)	359 (34)	0.14	0.51	0.94
	High (0.31–2.8)	415 (39)			
Tetracycline (parenteral)	No (0)	350 (33)			
	Medium (≤0.1)	408 (38)	0.36	0.32	0.80
	High (0.11–1.4)	312 (29)			
Tetracycline (oral)	No	620 (58)	0.21	0.23	0.98
	Use	450 (42)			
Pleuromutilin (oral)	No	712 (67)	0.10	0.21	0.01
	Use	358 (33)			
Other antibacterials (oral)^a^	No	724 (68)	0.03	0.10	0.78
	Use	346 (32)			
Other antibacterials (parenteral)^b^	No (0)	322 (30)	0.18	0.11	0.74
	Medium (≤0.1)	447 (42)			
	High (0.11–1.96)	301 (28)			

*The study included 1,070 finisher production herds with 2,906,626 finishers (14,411 of which had a recorded tail-bite observation) sent for slaughter at the single largest Danish abattoir during 2015*.

*^a^Includes macrolide, extended-spectrum penicillin, amphenicol, colistin, lincosamide, lincospectin, and sulfamethoxazole–trimethoprim*.

*^b^Includes lincosamide, macrolide, pleuromutilin, amphenicols, combination-drugs, lincospectin, sulfamethoxazole–trimethoprim, and extended-spectrum penicillin*.

In the final multivariate logistic regression models, herd size had a significant association with the occurrence of tail biting among slaughtered finishers (Table 3; *p* < 0.001), and with osteomyelitis (Table 4; *p* = 0.014) and hindquarter abscesses (Table 5; *p* < 0.001) among pigs with tail-bite observations. Small herds had increased odds of tail biting compared with medium-sized and large herds (Table 3). While the odds of osteomyelitis and hindquarter abscesses among pigs with tail-bites and associated with large herds were significantly smaller than those associated with medium-sized herds, there was no significant difference between small- and medium-sized herds. There was also a borderline-significant (*p* = 0.055) increased odds of osteomyelitis in finishers with tail-bite lesions that were treated orally with other antibacterials (Table 4), while the use of oral pleuromutilin was significantly negatively associated with the odds of hindquarter abscesses (Table 5; *p* = 0.022).

**Table 3:** Final multivariate logistic regression model of risk factors affecting the number of finishers with a tail-biting observation out of the total number of slaughtered finishers.

Risk factors		Estimate	SE	OR [CI_95%_]	*p*-Value^a^
Intercept		−5.518	0.047		
Herd size	Small (200–1,000)	0.232	0.072	1.26 [1.09; 1.45]	<0.001
	Medium (1,001–2,000)	0 (reference)	–	1	
	Large (2,001–12,000)	−0.083	0.077	0.92 [0.79; 1.07]	

*Herd was used as a random effect with an estimated SD of 0.899. Medium-sized herds (1,001–2,000 finishers) were the most frequently observed category, and was therefore used as reference*.

*^a^p-Values are based on the likelihood ratio tests*.

**Table 4:** Final multivariate logistic regression model of risk factors affecting the number of osteomyelitis from 14,411 finishers with a tail lesion registered at slaughter.

Risk factors		Estimate	SE	OR [CI_95%_]	*p*-Value^a^
Intercept		−2.477	0.066		
Other antibacterials (oral)	No	0 (reference)	–	1	0.055
	Use	0.153	0.079	1.16 [1.00; 1.36]	
Herd size	Small (200–1,000)	−0.074	0.097	0.93 [0.77; 1.12]	0.014
	Medium (1,001–2,000)	0 (reference)	–	1	
	Large (2,001–12,000)	−0.252	0.087	0.78 [0.65; 0.92]	

*Herd was used as a random effect with an estimated SD of 0.427. Herd size denotes the number of finishers registered in the herd during 2015. Medium-sized herds (1,001–2,000 finishers) were the most frequently observed category, and was therefore used as reference*.

*^a^p-Values are based on the likelihood ratio tests*.

**Table 5:** Final multivariate logistic regression model of risk factors affecting the number of hindquarter abscesses from 14,411 finishers with a tail lesion registered at slaughter.

Risk factors		Estimate	SE	OR [CI_95%_]	*p*-Value^a^
Intercept		−2.050	0.055		
Pleuromutilin (oral)	No	0 (reference)	–	1	0.022
	Use	−0.159	0.070	0.85 [0.74; 0.98]	
Herd size	Small (200–1,000)	0.003	0.082	1.00 [0.85; 1.18]	
	Medium (1,001–2,000)	0 (reference)	–	1	<0.001
	Large (2,001–12,000)	−0.316	0.075	0.73 [0.63; 0.84]	

*Herd was used as a random effect with an estimated SD of 0.107. Herd size denotes the number of finishers registered in the herd during 2015. Medium-sized herds (1,001–2,000 finishers) were the most frequently observed category, and was therefore used as reference*.

*^a^p-Values are based on the likelihood ratio tests*.

**Figure 2:**
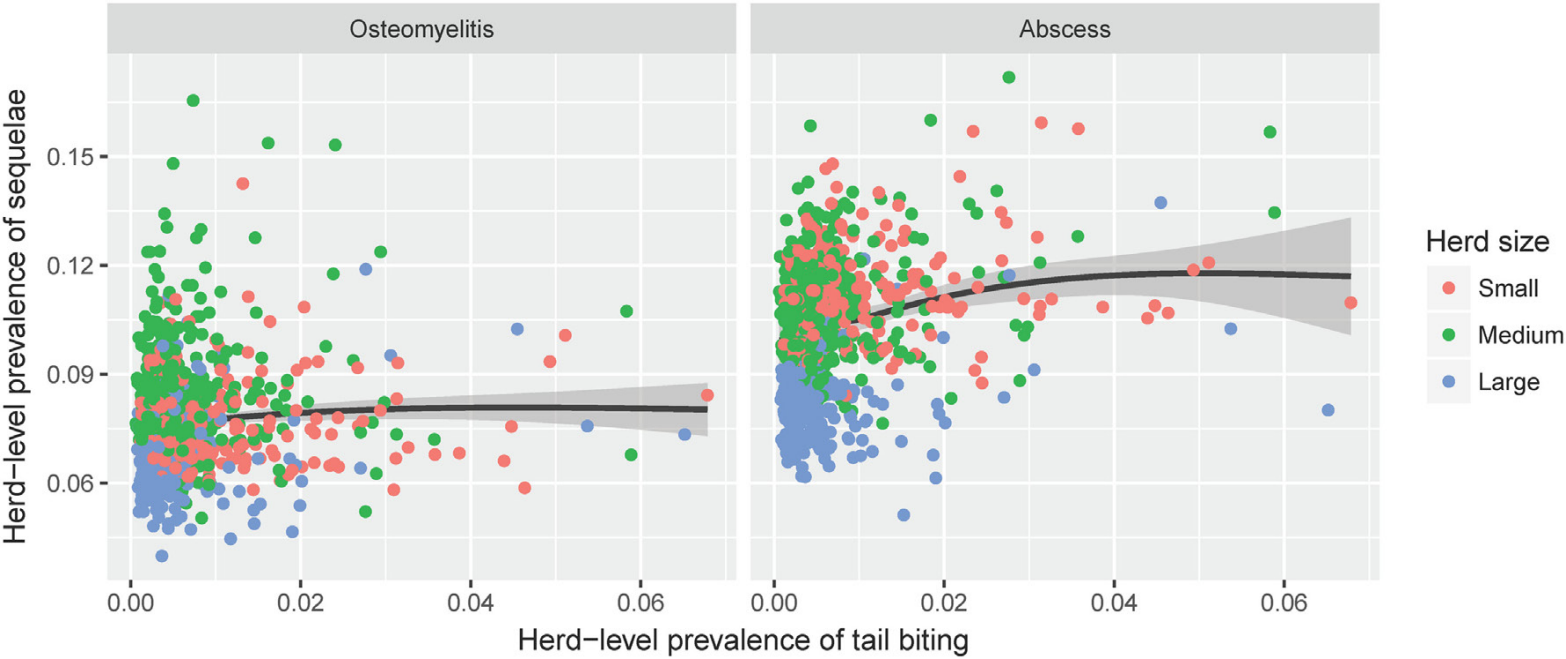
The estimated farm-level prevalence of tail-bite lesions among all slaughter pigs (*x*-axis) plotted against the estimated prevalence of sequelae (left: osteomyelitis, right: hindquarter abscesses) among pigs with signs of tail biting from the same farm (*y*-axis). Estimates were generated by the final logistic regression models for each of the three outcomes, taking into account all relevant fixed effects as well as the random effects structure within each model. The black line shows the estimated correlation, with gray shading indicating the 95% confidence interval.

It is possible to extract the herd-level prevalence estimates from the mixed effects models described above, taking into account the relevant risk factors as well as the estimated random effect for each herd. Based on these estimates, the relationship between the predicted herd-level prevalence of tail biting and that of related sequelae among pigs with tail-bite lesions from the same herd is shown in Figure 2. A large proportion of the predicted variation among herds is due to the random effect of herd relative to the fixed effect of herd size. There is a positive relationship between the predicted overall prevalence of tail biting and the proportion of pigs with tail-bite lesions that also have abscesses. This indicates that those farms with higher recorded rates of tail biting also have disproportionately more frequent observations of hindquarter abscesses. However, a similar relationship for osteomyelitis is not apparent.

## Discussion

### Antibacterial Use

The results of this study partly confirm our hypothesis, in that the use of first-choice antibacterials (oral pleuromutilins) was negatively associated with the proportion of hindquarter abscesses found in finishers with a tail-bite lesion at slaughter. Although borderline-significant, antibacterial use for purposes other than tail biting indicated a positive association with the proportion of osteomyelitis in finishers with a tail-bite lesion at slaughter. Macrolides, which are mainly used for gastrointestinal disorders (22), accounted for the largest group of other oral antibacterials (89%). The difference in association between antibacterial treatment and both osteomyelitis and hindquarter abscesses may be attributed to clinical challenges in treatment, in particular of osteomyelitis as a result of tail biting in finishing pigs. It is crucial to initiate treatment early, before osteomyelitis develops, because antibacterials generally have poor bone penetration, particularly penicillins, with a median bone-to-plasma concentration ratio of 0.16 (23). In addition, the increased osseous pressure and thrombosed vessels complicate treatment, while bacteria may even persist in sequesters formed in the chronic stages of infection (24). The challenges associated with osteomyelitis treatment are illustrated by the exceptionally long recommended treatment duration in human medicine of 4–6 weeks (24). To our knowledge, there have been no studies on the optimal treatment time for tail biting in finishing pigs to date. However, it can be assumed that even mild lesions of the skin may serve as entrance sites for potential pyogenic pathogens.

### Management

In addition to antibacterials, herd size was significantly associated with both of the tail-bite-related sequelae examined (Tables 4 and 5): osteomyelitis and hindquarter abscesses were both less likely to occur among finishers with tail-bite lesions from large herds (2,001–12,000 finishers) compared with medium-sized herds (200–2,000 finishers). The finding of similar risk factors for osteomyelitis and hindquarter abscesses is not unexpected, as the two outcomes were positively correlated (*p* < 0.001). One potential explanation for the significance of herd size is that large herds may have newer facilities and more experienced staff than small herds, which may reduce the risk of tail biting developing into severe cases with a higher risk of sequelae (3, 25).

As previously mentioned, management plays an essential role in the development of tail-biting behavior (2) and in the progression of injury, depending on whether or not the farmer manages to stop the behavior before it develops into a severe tail-biting outbreak. It can be challenging for the farmer to identify the individual pigs responsible for the tail biting, as it requires a high degree of awareness within the shed and is therefore highly dependent on management factors. The impact of the herd is underlined by the large differences in the herd prevalence of sequelae, with a small number of herds being responsible for the majority of cases. Tail biting is a multifactorial condition, and preventive initiatives, including reduced pig density and enrichment, are of utmost importance in keeping the herd prevalence to a minimum. As this study was based on register data, it was not possible to evaluate the effect of management-related factors. However, we restricted the study population to finisher-only herds, excluding herds with sows and/or weaners in order to limit variation in management procedures. Compared with integrated herds, we expect pure finisher herds to be less influenced by changes in productivity in the sow unit due to the introduction of animals from external sources. Through regular purchase of all pigs on the herd, we expect stocking density to be relatively consistent between finisher herds.

### Register-Based Data

Further caveats are also warranted for interpreting the results of this purely register-based study. Most importantly, we cannot necessarily conclude that there is a causal relationship within the associations that we have demonstrated. For example, it is far more likely that antibacterial treatment will be predominantly administered in groups of animals with a higher prevalence of disease, as opposed to the use of antibacterials increasing the prevalence of disease. This reverse causality could partially negate the effect of antibacterials reducing disease prevalence if given to randomly assigned groups. Furthermore, there are a number of uncertainties relating to the matching of databases. Slaughter remarks are registered at the individual pig level, while antibacterial purchase is registered at herd level within VetStat. This means that we have no information on treatments for individual animals. Instead, antibacterial use is quantified as the expected number of animals treated at herd level, based on a number of assumptions. Likewise, we have no information on whether finishers with tail lesions have been treated, or for which clinical indication they potentially received treatment. Each antibacterial purchase registered in VetStat has an associated intended indication. However, this indication does not necessarily represent the actual usage. Furthermore, the indication concerning tail biting, as recorded in VetStat, is broad, covering “locomotor, CNS, and skin diseases.” For this study, we therefore chose to specify treatments targeting tail biting purely based on administration route and active substances according to the official treatment guidelines and discussions with specialized practicing pig veterinarians.

Another challenge in matching VetStat and meat inspection data comes from the delay between the onset of clinical disease and eventual observation at slaughter, as well as the variable time lag between prospective antibacterial purchase and actual antibacterial use. Neither of these temporal issues could be addressed in this study, but we assume that the antibacterial treatment incidence as well as the proportion of tail-bite-related sequelae is relatively consistent over time.

### Meat Inspection Data

For this study, it would have been relevant to compare the prevalence of tail-biting-related sequelae in finishers with tail lesions to those of finishers with no registered tail bites. However, as pigs with tail-bite lesions are exposed to a pyemia investigation at the abattoir (6), the likelihood of identifying pyemic processes increases. Due to this discrepancy in *post-mortem* evaluation causing a selection bias, a comparison of finishers with and without tail lesions was not performed.

A large variation has previously been reported in recordings among Danish abattoirs (26), which makes it challenging to compare results. We therefore chose to restrict the study to one single abattoir. In addition, slaughter remarks have a generally low sensitivity (26). Prior studies have found the prevalence of tail lesions recorded at slaughter to be two to four times lower than the true prevalence (9). In this study, we found a tail-biting prevalence of 0.5%, which is somewhat lower than the figure of 1.26% as found in a prior clinical study by Petersen et al. (8) as well as the figure of 0.84% as found in a *post-mortem* study by Hansen and Agerley (7). The two studies by Petersen et al. (8) and Hansen and Agerley (7) were based on clinical observations performed during the period 1999–2001, and registrations on slaughter carcasses in 2004, respectively. Since then, there has been an increased focus on initiatives to prevent tail biting (27), which may explain the lower prevalence presented in our study. Another explanation of the observed discrepancies in prevalence may be found in the differences between clinical observation and slaughter remarks, as mentioned in Section “Introduction.” When evaluating this prevalence in an international context, it should also be noted that tail docking is practiced for the majority of Danish finishers.

## Conclusion

Results of this study found the use of oral pleuromutilin to be significantly negatively associated with the odds of hindquarter abscess observations at slaughter in finishers with tail-bite lesions. The apparent differences in association between antibacterial treatment and tail-biting-related sequelae may be partly explained by clinical challenges in treatment, particularly of infections in the bone. Herd size was also significantly associated with the prevalence of both osteomyelitis and hindquarter abscesses in finishers with tail lesions registered at the time of slaughter. This highlights the multifactorial etiology of tail-biting, and points toward the importance of management procedures. This study was purely register-based, and so the associations presented here should be interpreted with caution. An intervention study to further examine the effect of antibacterial treatment on tail-biting-related injuries would be highly beneficial in order to validate our findings.

## Author Contributions

MF and AB initiated the study, MF, MD, and AB designed the study, MF and CB performed data management, MF and MD analyzed the data, MF, MD, CB, HS, and AB interpreted the results, and MF drafted the manuscript. All authors read and approved the final manuscript.

## Conflict of Interest Statement

The authors declare that the research was conducted in the absence of any commercial or financial relationships that could be construed as a potential conflict of interest.
